# Supplementary material for: Acral Melanoma Is Infiltrated with cDC1s and Functional Exhausted CD8 T Cells Similar to the Cutaneous Melanoma of Sun-Exposed Skin
Source: Int J Mol Sci. 2023 Mar 1;24(5):4786. doi: 10.3390/ijms24054786 (PMC10003718; doi:10.3390/ijms24054786)
Supplement: Supplementary file 1 [file ijms-24-04786-s001.zip › ijms-2068629-supplementary.pdf]

Supplementary material

Supplementary Tables

Patient	Age	Sex	Melanoma type	Stage	Breslow	Primary lesion	Sun exposure
1	66	M	NACM	I	2	Scalp	High
2	53	F	NACM	I	0.8	Leg	High
3	81	M	NACM	III	23	Back	High
4	74	F	NACM	II	3	Leg	High
5	70	M	NACM	I	0.3	Scalp	High
6	78	F	NACM	I	0.25	Malar region	High
7	76	M	NACM	I	7	Face	High
8	67	F	NACM	I	0.1	Lower eyelid	High
9	68	M	AM	II	3	Sole	Low
10	79	M	AM	I	1.1	Toe	Low
11	73	F	AM	III	4	Sole	Low
12	59	F	AM	I	0.6	Sole	Low
13	72	M	AM	I	NR	Sole	Low
14	85	M	AM	I	0.3	Sole	Low
15	67	F	AM	II	12	Sole	Low
16	85	F	AM	I	2	Sole	Low
17	61	M	AM	III	7	Sole	Low
18	66	F	AM	II	11	Sole	Low
19	50	F	AM	III	11	Sole	Low
20	75	F	AM	III	4	Sole	Low
21	68	M	AM	II	12	Sole	Low
22	51	F	AM	IV	12	Sole	Low
23	70	M	AM	I	0.3	Sole	Low
24	41	M	AM	II	2.2	Sole	Low
25	78	M	AM	III	13	Sole	Low
26	37	F	AM	I	NR	Toe	Low
27	63	M	AM	III	13	Toe	Low
28	61	F	AM	I	NR	Toe	Low
29	74	M	AM	III	2	Finger	Low
30	73	F	AM	II	23	Toe	Low
31	65	F	AM	II	13	Sole	Low
32	83	F	AM	II	3	Sole	Low
33	77	M	AM	III	13	Toe	Low
34	70	F	AM	III	16	Finger	Low
35	59	F	AM	II	4	Finger	Low
36	65	M	AM	II	6	Toe	Low
37	53	M	AM	I	0.4	Sole	Low
38	56	F	AM	II	2	Heel	Low
39	81	M	NACM	IV	NR	Shoulder	High
40	55	M	NACM	IV	NR	Dorsum of hand	High
41	71	M	NACM	IV	NR	Chest	High
42	66	F	NACM	IV	NR	Behind the ear	High
43	64	F	NACM	IV	NR	Leg	High

Supplementary Table S1. Demographic and clinical characteristics of NACM and AM patients. Description of age, sex (F-Female and M- Male), Stage and Breslow index for every patient in the cohort. Primary lesions were considered to establish the amount of sun exposition of the skin as well as to classify patients as NACM and AM. The rows in blue represent the five additional advanced NACM samples taken from patients of other IMSS and private hospitals that were used for the analysis of Figures 2-6.

ANTIBODY	BRAND	CLONE	CAT NUMBER	ASSAY
CD45-PE/Cy7	Biolegend	H130	304016	Flow Cytometry
CD45RO-PE/Dazzle94	Biolegend	UCHL1	304247	Flow Cytometry
CD8-APC/Cy7	Biolegend	SK1	344713	Flow Cytometry
CD4-BV650	Biolegend	OKT4	317436	Flow Cytometry
CD69-BV750	Biolegend	FN50	310953	Flow Cytometry
PD-1-PE	Biolegend	EH12.2H7	329906	Flow Cytometry & IF
Live/Dead Fixable Violet	Invitrogen		L34955	Flow Cytometry
IFN-γ-BV510	Biolegend	4S.B3	502543	Flow Cytometry
KI-67-BV605	Biolegend	Ki-67	350522	Flow Cytometry
CD11c-purified	Abcam	EP1347Y	ab52632	IF
HLA-DR-purified	Invitrogen	YD1/63.4.10	MA1-70113	IF
BDCA3-purified	Abcam	PBS-01	ab6980	IF
HMB-15-Alexa Fluor 647	Biolegend	HMB-45	911506	IF
MART1-purified	Abcam	EPR20380	ab210546	IF
CD8-purified	Invitrogen	YTC182.20	MA1-81692	IF
PD-L1-APC	Biolegend	29E.2A3	329708	IF
IFN-γ-FITC	SONY	B27	3132520	IF
KI-67-purified	Invitrogen	SolA15	14-5698-82	IF

Supplementary Table S2. List of antibodies used to perform the flow cytometry or immunofluorescence (IF) assays.

Supplementary Figures

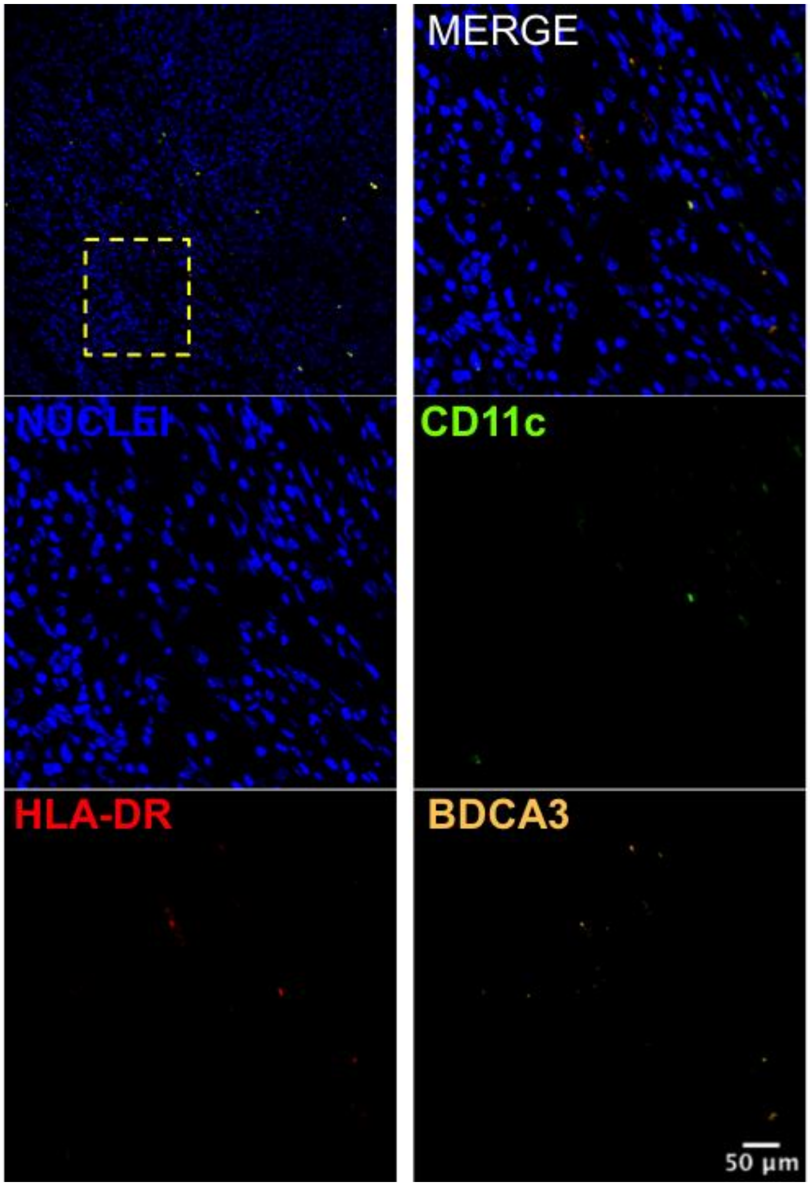

Supplementary Figure S1. DCs autofluorescence staining control. Tissue was processed in the same way as the experimental staining, except no primary antibodies anti-CD11c, -HLA-DR and -BDCA3 were added. Top panels show the merged images at 20x (left) and a digital zoom of the selected area (right). Middle and low panels show the signal of each channel to provide a better appreciation of the autofluorescence of each fluorochrome in the tissue.

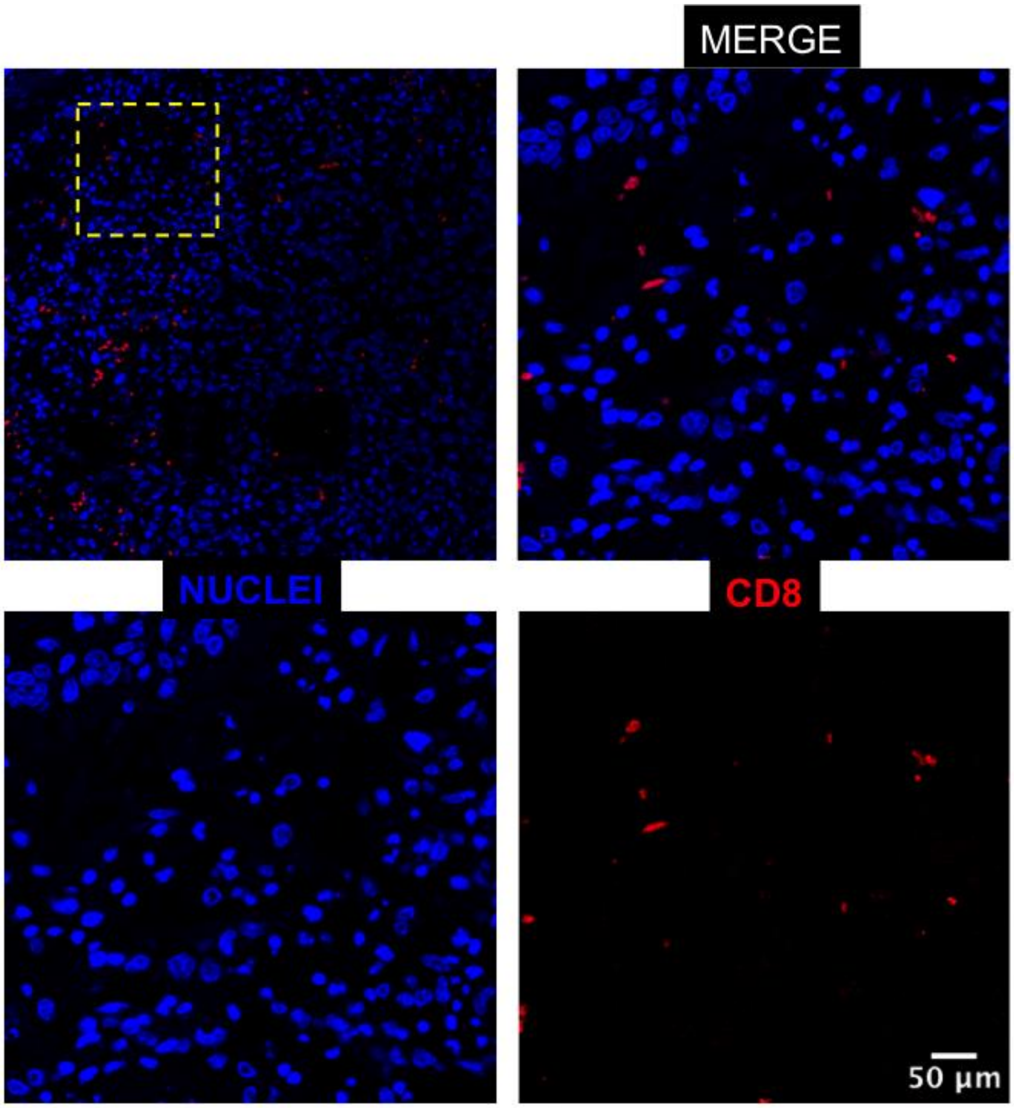

Supplementary Figure S2. CD8 autofluorescence staining control. Tissue was processed in the same way as the experimental staining, except no primary antibody (anti-CD8) was added. Top panels show the merged images at 20x (left) and a digital zoom of the selected area (right). Low panels show the signal of each channel to provide a better appreciation of the autofluorescence of each fluorochrome in the tissue.

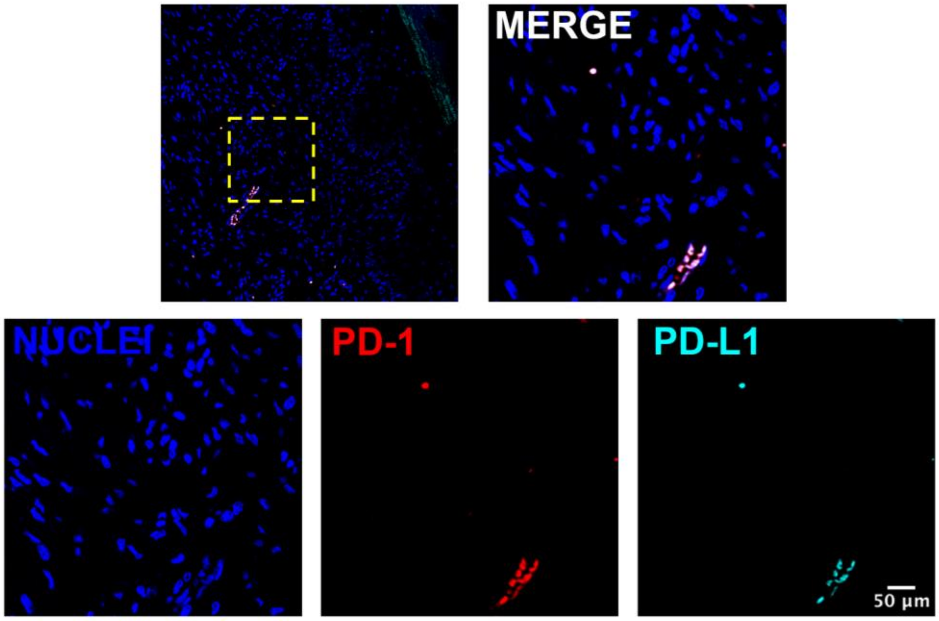

Supplementary Figure S3. PD-1 and PD-L1 autofluorescence staining control. Tissue was processed in the same way as the experimental staining, except no antibodies anti-PD-1 and -PD-L1 were added. Top panels show the merged images at 20x (left) and a digital zoom of the selected area (right). Low panels show the signal of each channel to provide a better appreciation of the autofluorescence of each fluorochrome in the tissue.

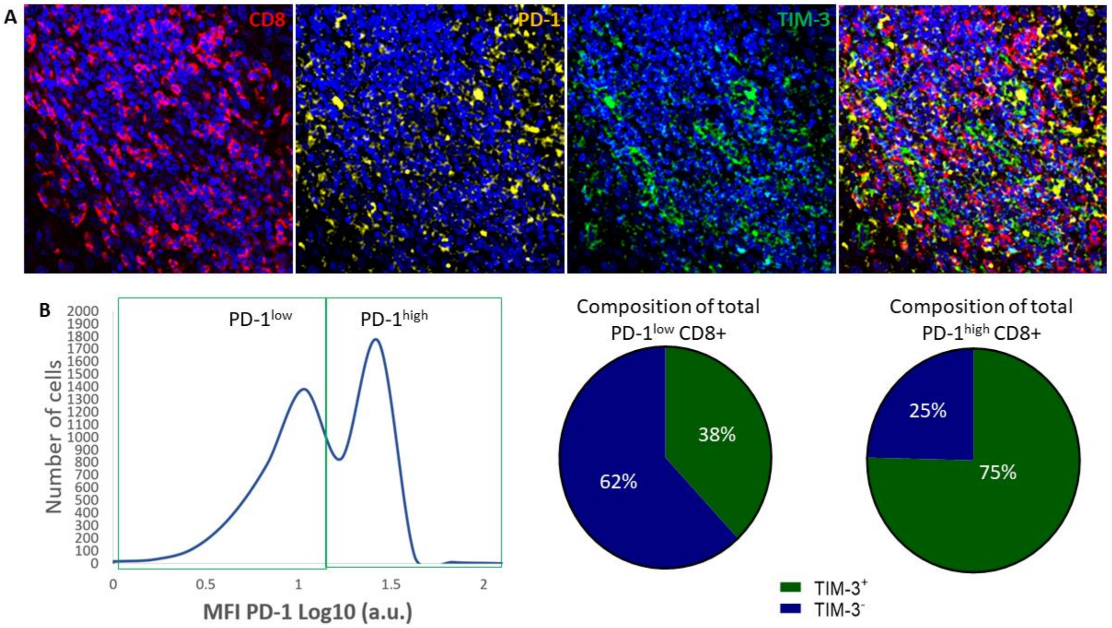

Supplementary Figure S4. TIM-3 expression on PD-1 low and high CD8 T cells. A) Representative immunofluorescence image stained for CD8 (red), PD-1 (yellow), TIM-3 (green) of acral melanoma patients (n = 5). B) Histogram showing the regions where the pie charts were obtained (green squares). The left pie chart represents the percentage of cells that are TIM-3 positive or negative on PD-1 low CD8+ cells fraction. The right pie chart represents the percentage of cells that are TIM-3 positive or negative on PD-1 high CD8+ cells fraction.

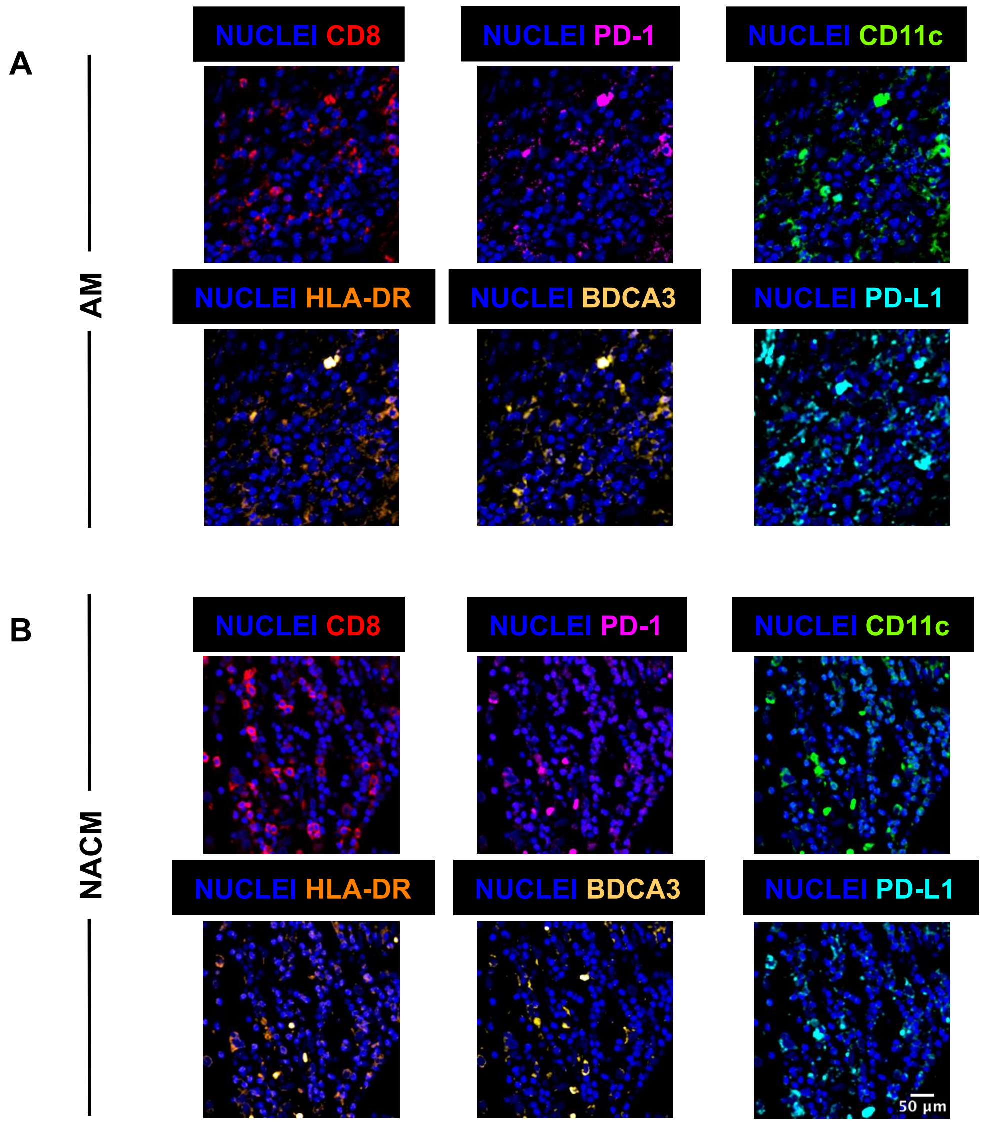

Supplementary Figure S5. Multiplexed Immunofluorescence of PD-1 and PD-L1 expression in CD8 T cells and dendritic cells. Representative images of the reconstructions of every analyzed mark for (**A)** AM and (**B)** NACM patients. CD8 (red), PD-1 (magenta), CD11c (green), HLA-DR (orange), BDCA3 (yellow) and PD-L1 (cyan).

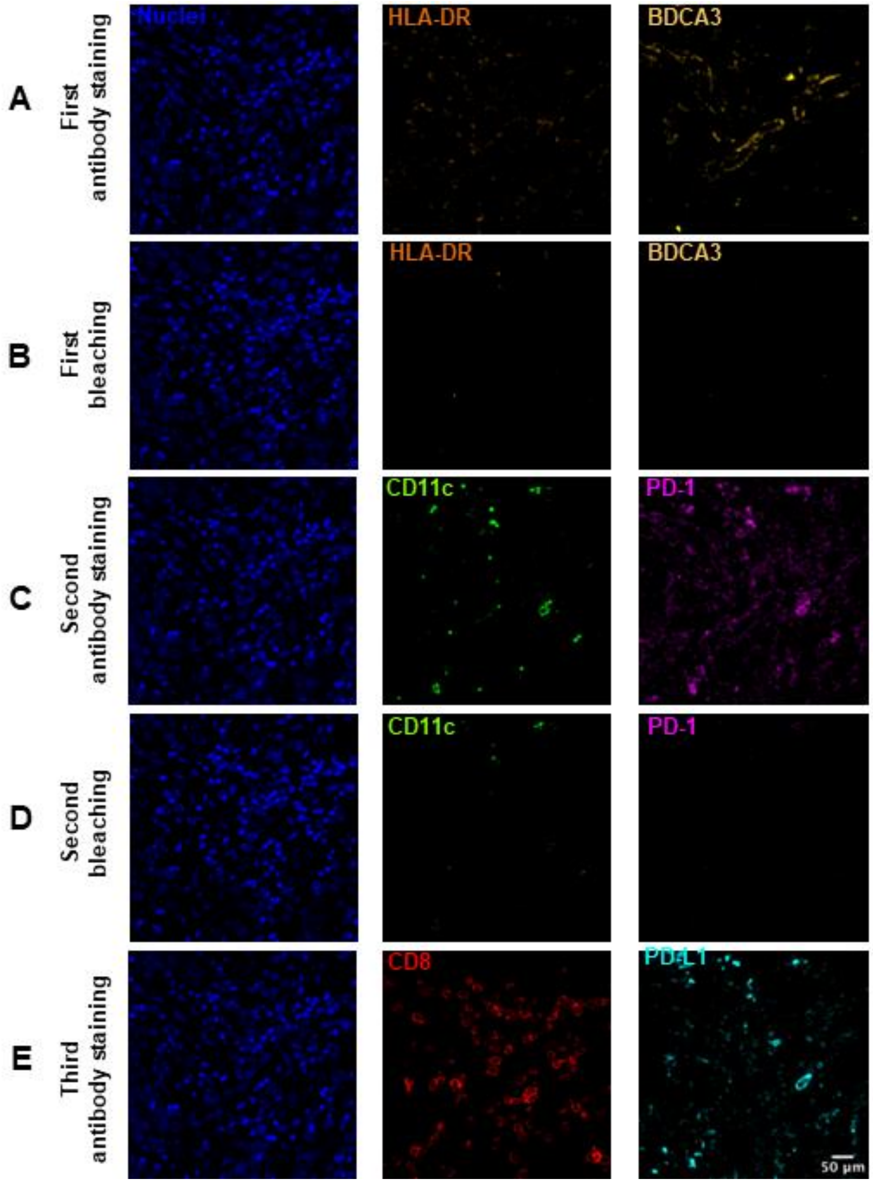

Supplementary Figure S6. Signal removal upon each bleaching cycle for the multiplexed immunofluorescence of PD-1 and PD-L1 expression in CD8 and dendritic cells. Representative images acquired after every staining and bleaching cycle. First staining (A) included anti-HLA-DR (orange) and anti-BDCA3 (yellow), and bleaching cycle (B) in which marks in (A) are not further detectable. Second staining (C) included anti-CD11c (green) and anti-PD-1 (magenta) and bleaching cycle (D) in which marks in (C) are not further detectable. E) Final staining with anti-CD8 (red) and anti-PD-L1 (cyan).

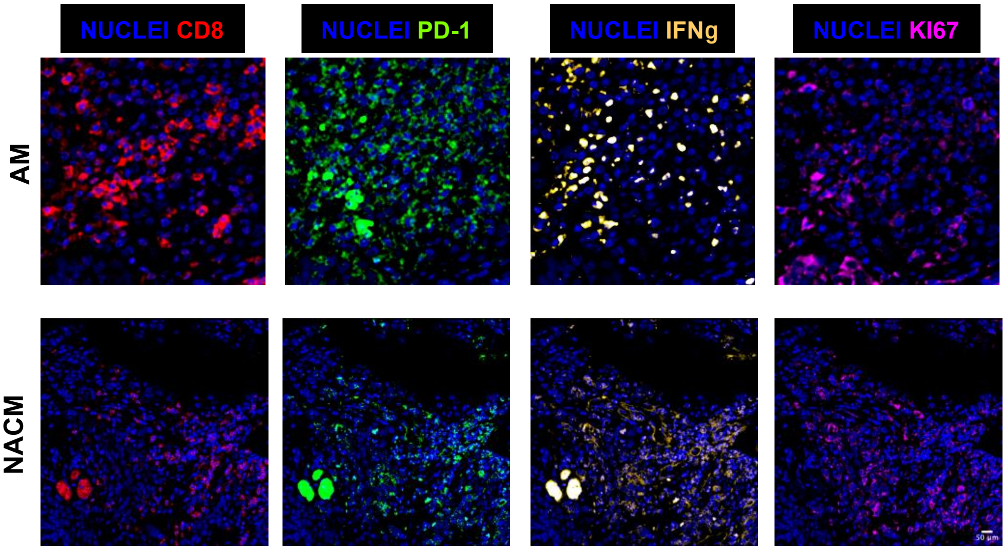

Supplementary Figure S7. Multiplexed immunofluorescence to assess the expression of markers of function in PD-1⁺ CD8 cells. Representative images of the reconstructions of every analyzed staining for AM and NACM patients. CD8 (red), PD-1 (green), IFN-γ (yellow) and KI-67 (magenta).

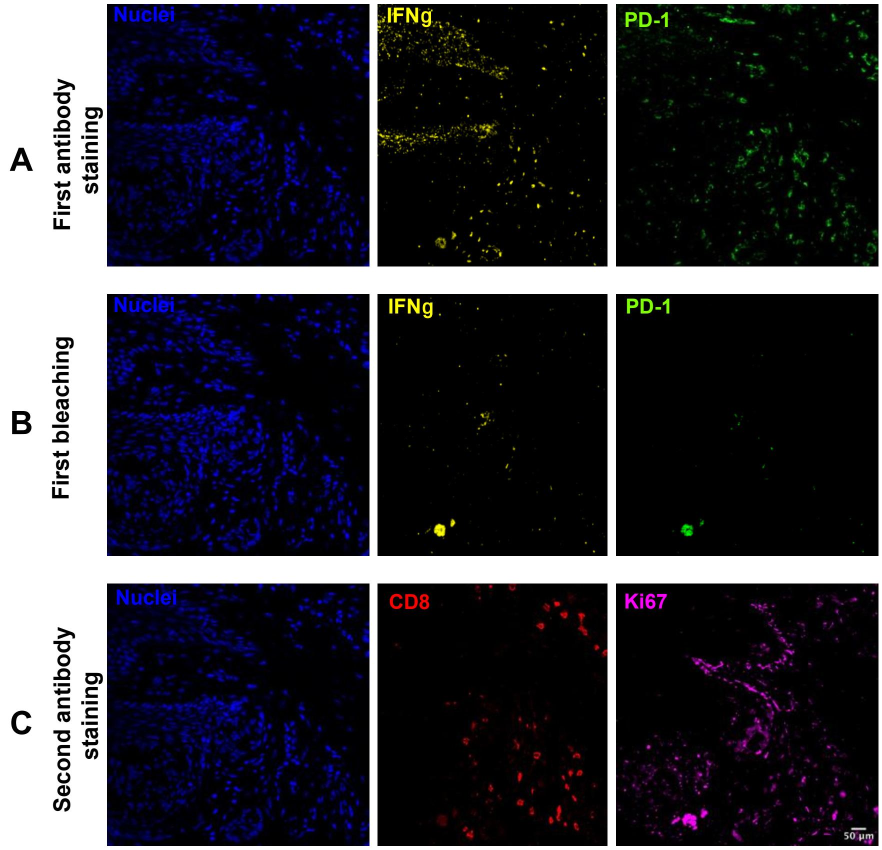

Supplementary Figure S8. Signal removal upon each bleaching cycle for the multiplexed immunofluorescence of PD-1 and PD-L1 expression on CD8 PD-1 cells. Representative images acquired after every staining and bleaching cycle. First staining (A) included anti-IFN-γ (yellow) and anti-PD-1 (green), and bleaching cycle (B) in which marks in (A) are not further detectable. C) Final staining with anti-CD8 (red) and anti-KI-67 (magenta).

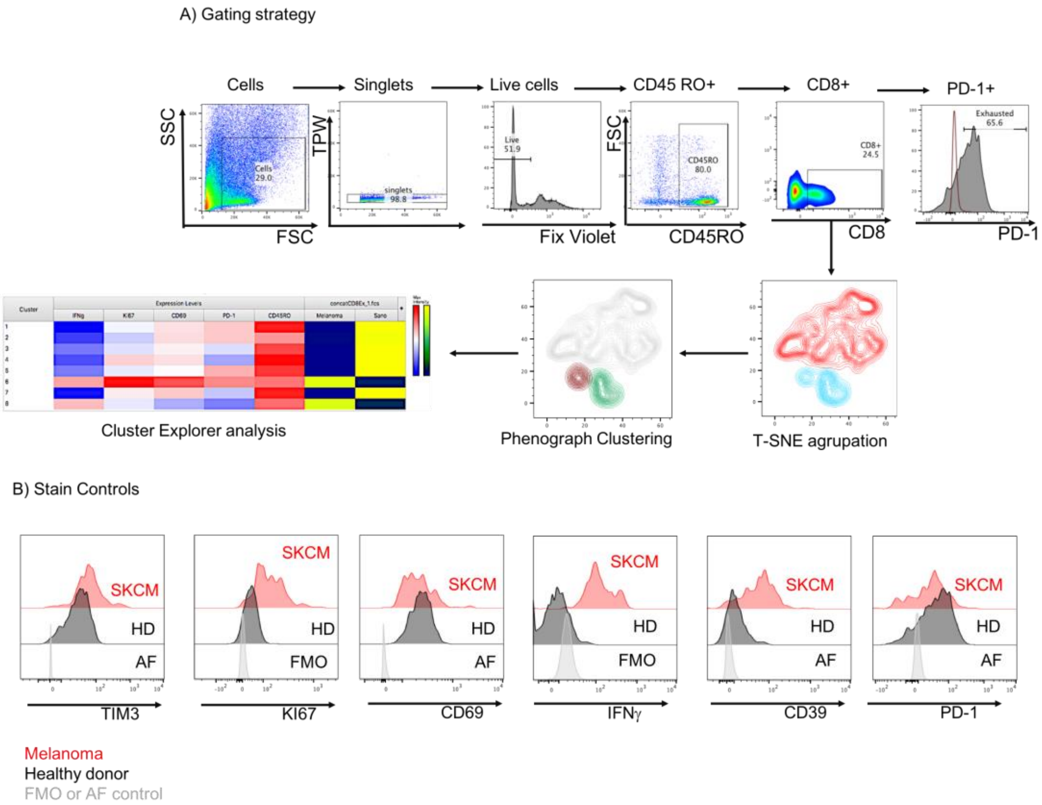

Supplementary Figure S9. A) Gating strategy for the flow cytometry data of Figure 7. Lymphoid cells were gated from SSC and FSC parameters, followed by singlets selection, live cells (viability dye negative), memory cells (CD45RO+) and CD8+ cells. Histograms were obtained from CD45RO+ CD8 T cells as well as the T-SNE plot. B) Staining controls were used to stablish the positive thresholds for each marker. Autofluorescence (AF) was used for membrane antigens (TIM-3, CD69, CD39 and PD-1), while fluorescence minus one (FMO) controls were used for intracellular and nuclear antigens (KI-67 and IFN-.)γ Red histograms (melanoma), black histograms (healthy donor) and grey histograms (staining controls)
